# Postoperative pain management in patients undergoing posterior spinal fusion for adolescent idiopathic scoliosis: a narrative review

**DOI:** 10.1186/s13013-018-0165-z

**Published:** 2018-09-12

**Authors:** Hiroyuki Seki, Satoshi Ideno, Taiga Ishihara, Kota Watanabe, Morio Matsumoto, Hiroshi Morisaki

**Affiliations:** 1 Department of Anesthesiology, Keio University School of Medicine, 35 Shinanomachi Shinjuku-ku, Tokyo, 160-8582 Japan; 2 Department of Orthopaedic Surgery, Keio University School of Medicine, 35 Shinanomachi Shinjuku-ku, Tokyo, 160-8582 Japan

**Keywords:** Analgesia, Pain management, Scoliosis, Spine surgery

## Abstract

Posterior spinal fusion for adolescent idiopathic scoliosis is one of the most invasive surgical procedures performed in children and adolescents. Because of the extensive surgical incision and massive tissue trauma, posterior spinal fusion causes severe postoperative pain. Intravenous patient-controlled analgesia with opioids has been the mainstay of postoperative pain management in these patients. However, the use of systemic opioids is sometimes limited by opioid-related side effects, resulting in poor analgesia. To improve pain management while reducing opioid consumption and opioid-related complications, concurrent use of analgesics and analgesic modalities with different mechanisms of action seems to be rational. The efficacy of intrathecal opioids and nonsteroidal anti-inflammatory drugs as components of multimodal analgesia in scoliosis surgery has been well established. However, there is either controversy or insufficient evidence regarding the use of other analgesic methods, such as continuous ketamine infusion, perioperative oral gabapentin, acetaminophen, continuous wound infiltration of local anesthetics, a single dose of systemic dexamethasone, and lidocaine infusion in this patient population. Moreover, appropriate combinations of analgesics have not been established. The aim of this literature review is to provide detailed information of each analgesic technique so that clinicians can make appropriate choices regarding pain management in patients with adolescent idiopathic scoliosis undergoing posterior spinal fusion.

## Background

Adolescent idiopathic scoliosis (AIS) is a three-dimensional deformity of the spine defined as a lateral curvature of the spine in the coronal plane of more than 10° with no known cause and a predilection for adolescent girls [1]. The degree of curvature is determined by the Cobb angle (formed by lines drawn along the superior endplate of the superior vertebra and the inferior endplate of the inferior vertebra). Surgical correction is indicated for AIS with severe deformity (Cobb angle > 45°), which can decrease the vital capacity of the lungs and interfere with everyday activity [2]. Posterior spinal fusion (PSF), which accounts for over 90% of surgical procedures performed for the correction of scoliosis, causes severe postoperative pain because of extensive dissection of the skin, subcutaneous tissues, bones, and ligaments. Innervation of these structures is via the posterior rami of the spinal nerves connected to the sympathetic and parasympathetic nerves, and various noxious stimuli, including mechanical irritation, compression, or postoperative inflammation, can cause pain [3]. In addition, the majority of patients with AIS who undergo PSF are young women, who are particularly susceptible to pain [4, 5]. The causes of pain are complex, so the management of pain after scoliosis surgery is challenging. Inadequate pain management may not only diminish patients’ satisfaction, but also result in delayed postoperative recovery, ambulation, discharge, and even persistent postoperative pain [6]. Historically, intravenous (IV) patient-controlled analgesia (PCA) with opioids has been the mainstay of pain management after scoliosis surgery. However, opioid-related adverse effects, such as nausea and vomiting, remain problematic for the majority of patients [7]. A recent trend has been to use more than one class of analgesic agent or technique concurrently to improve analgesia while reducing opioid consumption and opioid-related complications. Since the concept of multimodal analgesia was first introduced in the treatment of postoperative pain [8], its value has been well described, especially in joint arthroplasty [9]. Over the last few decades, various analgesics or analgesic modalities have been proposed as components of multimodal analgesia for scoliosis surgery, but there is either controversy or insufficient evidence regarding the use of these agents and modalities, so no optimal regimen has been established. The aim of this literature review is to provide detailed information on each analgesic technique so that clinicians can make choices regarding pain management in patients undergoing PSF for AIS.

## Methodology

We searched the PubMed, Web of Science, and Cochrane Library to identify articles on postoperative analgesia in patients undergoing correction surgery for AIS using the keywords “scoliosis,” “analgesia,” “postoperative,” and “children.” One hundred and sixty-two potentially eligible records were retrieved. Articles were selected based on their relevance to the topic and limited to humans and publication in the English language up to November 2017. Case reports, case series, systematic reviews, meta-analyses, and scientific abstracts were excluded. Given that our focus was on postoperative pain management in patients undergoing PSF for AIS, articles that did not include PSF were excluded. After excluding 126 studies (63 duplicate publications, 10 review articles or meta-analyses, 10 case reports, 45 not relevant to the topic, 1 scientific abstract, 5 without clear details of the technique used, and 4 with inaccessible full text), 31 studies were included in the review. Additional references were identified from reference lists in the publications yielded by our initial search. This article was not intended to be a systematic review, so we did not apply the strict methodology recommended by PRISMA [10].

### Intravenous PCA

PCA allows patients to self-administer analgesics, mainly opioids, intermittently as needed. IV PCA has been used as the “gold standard” treatment in adults with moderate to severe pain. There is also evidence showing that PCA is safe and effective in children [11]. Morphine-based IV PCA is the most commonly used form of postoperative analgesia in patients undergoing PSF for AIS [12, 13]. Morphine is generally administered as an initial loading dose of 0.05–0.1 mg/kg before the end of surgery, with PCA settings of a bolus dose of 0.01–0.03 mg/kg, a lockout interval of 6–10 min, and a background infusion of 0.01–0.02 mg/kg/h [14–32] (Table 1). Some guidelines advise against the use of a background infusion in adults because of the potential for respiratory depression [33–35]. However, background infusion is widely used in children [36], who are considered to have a lower risk of respiratory depression [37, 38]. A study of the efficacy and safety of PCA in patients with AIS [14] found no difference between PCA alone and PCA with background infusion with regard to the postoperative use of morphine, side effects, or patient satisfaction and concluded that background infusion did not provide clinically significant advantages over intermittent bolus doses of morphine. The main adverse effects of IV PCA with morphine are nausea and vomiting (20–80%) and pruritus (11–47%), but clinically significant respiratory depression is rare. One retrospective study investigated the adverse effects of postoperative continuous morphine infusion without PCA (the infusion rate was titrated by increments of 0.005–0.01 mg/kg/h to keep visual analog scale (VAS) pain scores at 5 or less, with a maximum infusion rate of 0.06 mg/kg/h) in combination with intrathecal morphine [39]. The incidences of postoperative nausea and vomiting (PONV) (13.3%) and pruritus (4.1%) were similar to those for PCA, and no cases of respiratory depression or admissions to the pediatric intensive care unit were reported. In addition to morphine, oxycodone (0.025 mg/kg every 10 min on demand with a 4-h maximum of 0.3 mg/kg) was also used for PCA [32].

**Table 1:** Regimens for intravenous patient-controlled analgesia reported in the literature

First author Journal Year Type of study Total sample size	Loading	PCA bolus	Lockout interval	Basal infusion
Weldon [14] *Clin J Pain* 1993 RCT 54	None	Morphine sulfate 0.03 mg/kg (PCA bolus alone) or 0.02 mg/kg (PCA bolus + basal infusion)		None (PCA bolus alone) or morphine sulfate 0.02 mg/kg/h (PCA bolus + basal infusion)
Beaulieu [15] *Int Orthop* 1996 Observational 100	Morphine 114.5 μg/kg	Morphine 24.8 μg/kg	9.9 min	None
Cassady [16] *Reg Anesth Pain Med* 2000 RCT 33	None	Morphine sulfate 0.02 mg/kg	8-min and 4-h limit of 0.2 mg/kg	None
Munro [17] *Can J Anaesth* 2002 RCT 35	Morphine 0.05–0.1 mg/kg	Morphine 0.02 mg/kg	No description	Morphine 0.01 mg/kg/h
Sucato [18] *Spine* (*Phila Pa 1976*) 2005 Retrospective 613	None	Morphine 0.02–0.03 mg/kg or meperidine 0.2–0.3 mg/kg	7–12 min with a limit of 4–5 injections/h	± Morphine 0.015 mg/kg/h or meperidine 0.15 mg/kg/h
Crawford [19] *Anesth Analg* 2006 RCT 30	Morphine 100 μg/kg	Morphine 20 μg/kg	6 min	Morphine 10 μg/kg/h
Gauger [20] *J Pediatr Orthop* 2009 RCT 67	None	Hydromorphone 2 μg/kg	10-min and 4-h limit of 20 μg/kg	Hydromorphone 2 μg/kg/h
Milbrandt [21] *Spine* (*Phila Pa 1976*) 2009 Retrospective 136	None	Morphine 0.01–0.02 mg/kg	6–10 min with a limit of 4–5 injections/h	± Morphine 0.02 mg/kg/h
Sadhasivam [22] *J Clin Anesth* 2009 Retrospective 131	None	Morphine 20 μg/kg	7 min	Morphine 10 μg/kg/h
Wade [23] *Spine Deform* 2015 Retrospective 196	None	Morphine 10 μg/kg	10 min	Morphine 10 μg/kg/h
Mayell [24] *Pediatr Anaesth* 2014 RCT 35	None	Morphine 20 μg/kg	6 min	Morphine 10 μg/kg/h for the first day
Klatt [25] *Spine* (*Phila Pa 1976*) 2013 RCT 66	Up to 0.2 mg/kg	Morphine 20 μg/kg	10 min with a limit of 4 demand doses/h	Morphine 20 μg/kg/h
Choudhry [26] *J Pediatr Orthop* 2017 Retrospective 127	None	Morphine 1.0 mg	10 min with a 4-h maximum dose of 0.3 mg/kg	Morphine 20 μg/kg/h
Perello [27] *Spine* (*Phila Pa 1976*) 2017 RCT 44	None	Morphine hydrochloride 20 μg/kg		Morphine hydrochloride 5 μg/kg/h
Pestieau [28] *Pediatr Anesth* 2014 RCT 50	None	Morphine 20 μg/kg	8 min	Morphine 20 μg/kg/h
Rusy [29] *Anesth Analg* 2010 RCT 59	None	Morphine 0.02 mg/kg	6 min and an hourly maximum of 0.12 mg/kg	Morphine 0.02 mg/kg/h
Engelhardt [30] *Anesth Analg* 2008 RCT 34	None	Morphine 20 μg/kg	6 min	Morphine 10 μg/kg/h
O’Hara [31] *Pediatr Anesth* 2004 RCT 31	None	Morphine 1 mg (BW > 50 kg) or 20 μg/kg (BW < 50 kg)	6 min with 10 possible doses/h	None
Hiller [32] *Spine* (*Phila Pa 1976*) 2012 RCT 36	None	Oxycodone 0.025 mg/kg	10-min and 4-h maximum of 0.3 mg/kg	None

*BW* body weight, *PCA* patient-controlled analgesia, *RCT* randomized controlled trial

In summary, IV PCA with morphine is the best established and most widely used analgesia technique for scoliosis correction surgery. However, considering the high incidence of minor complications, such as postoperative nausea and vomiting (PONV) and pruritus, other techniques should be considered for the combination with IV PCA to minimize the use of morphine.

### Epidural analgesia

Epidural analgesia is also widely used as an adjunct to IV PCA for postoperative analgesia in patients undergoing PSF for scoliosis [16, 20, 25, 31, 40–43]. However, no standard methods have been established. Although an epidural catheter is always placed by the surgeon prior to the closure of the surgical wound, the numbers and positions of the catheters inserted, the types and amounts of agents used, and the mode of administration vary widely in the literature (Table 2). Recent meta-analyses show that epidural analgesia is superior in analgesic effect when compared with IV PCA, making it possible to reduce the amount of analgesics used postoperatively [32, 33]. However, the success rate varies depending on the report. In a retrospective study that compared postoperative epidural analgesia (*n* = 413) and IV PCA (*n* = 200), the averages of all pain scores and scores at 2, 4, 6, 8, 12, 24, 36, and 48 h were significantly better in the epidural analgesia group. However, the need for temporary cessation and permanent discontinuation of pain management was greater in the epidural analgesia group than in the IV PCA group (12.3% vs 7.0% and 13.1% vs 0.0%, respectively) [18]. The most common reasons for temporary cessation of epidural analgesia in the 413 patients were respiratory depression (*n* = 30, 7.3%), neurologic changes (*n* = 13, 3.1%), and oversedation (*n* = 3, 0.7%). The neurologic changes were mild asymmetric differences in the sensation of light touch on the lower extremities that resolved after temporary cessation of the epidural infusion. The most common reasons for permanent discontinuation of epidural analgesia were uncontrollable pain (*n* = 33, 8.0%) and neurologic changes (*n* = 8, 2.1%). There were no permanent neurologic injuries. In a small randomized controlled trial that compared epidural analgesia (*n* = 19) and IV PCA (*n* = 19), seven patients (37%) in the epidural analgesia group experienced poor analgesia and required a change to IV PCA within 24 h, although the average and lowest pain scores on postoperative days 2 and 3 were significantly lower in the epidural group [20]. In contrast, an observational study that assessed 98 consecutive patients undergoing PSF who received epidural analgesia reported insufficient analgesia in only 3 patients [41]. In that study, there were no adverse events in 87 patients. The most common complications were hypotension (*n* = 3), insufficient analgesia (*n* = 3), and displacement of the epidural catheter (*n* = 2). The epidural catheter was removed on postoperative day 2 in one patient because of the development of a leak at the skin level. There was a further patient in whom a leak into the spinal space was suspected because of prolonged muscle weakness. It is likely that the efficacy and safety of epidural analgesia depend on the experience of health care providers. The frequency of the most common minor complications, such as PONV (in up to 85.7% of cases) and pruritus (in up to 42.9%), also varies depending on the report. Some studies have shown that epidural analgesia has a lower incidence of these adverse effects than IV PCA, while others have found no difference between the two techniques. There are some epidural analgesia-specific complications. In a randomized controlled study, 4 of 15 patients showed motor blockade of more than one (with a modified Bromage scale score of 2 in one patient and 3 in three patients) after receiving an initial bolus of epidural ropivacaine [40]. The blockade spontaneously resolves within 180 min but might interfere with the neurologic assessment. Thus, the neurologic function should be assessed before administering a loading dose of epidural analgesia. In another randomized controlled trial that compared epidural analgesia and IV PCA, urinary retention was observed in 3 of 22 patients in the epidural group. Although the incidence of urinary retention was not different between the two analgesic techniques, postoperative epidural analgesia has been reported to be associated with a higher incidence of urinary retention than IV PCA [44]. No epidural analgesia-related infections have been reported, but because of the small sample sizes in most of the studies, the risk of these infections is not completely excluded.

**Table 2:** Regimens for continuous epidural analgesia reported in the literature

First author Journal Year Type of study Total sample size	Catheter position	Loading	Continuous infusion	PCEA
Cassady [16] *Reg Anesth Pain Med* 2000 RCT 33	At the midpoint of the incision and advanced 3–5 cm cephalad	Bupivacaine 0.25% with epinephrine 1:200,000, 10 ml, 15 min before skin closure	Bupivacaine 0.125% (0.35 mg/kg/h) and fentanyl 2.5 μg/ml (0.7 μg/kg/h), 0.28 ml/kg/h, within 30 min of arrival in the PACU	None
Tobias [42] *Paediatr Anaesth* 2001 Observational 14	Upper catheter: at the T6–8 level with the tip directed cephalad to T1–4. Lower catheter: at the T12 level with the tip directed caudad to the L1–4 level.	Fentanyl 1 μg/kg and hydromorphone 5 μg/kg were diluted in 0.3 ml/kg of saline. Following placement, the upper and lower catheters were dosed with 0.1 ml/kg and 0.2 ml/kg of the solution, respectively. After tracheal extubation and neurologic examination, the catheters were dosed with 0.1 ml/kg of ropivacaine 0.2% (upper catheter) and 0.2 ml/kg of ropivacaine 0.2% (lower catheter)	Ropivacaine 0.1% and hydromorphone 10 μg/ml at 0.1 ml/kg/h into the upper catheter and 0.2 ml/kg/h into the lower catheter, started after the bolus doses of ropivacaine	None
Blumenthal [40] *Anesthesiology* 2005 RCT 30	Upper catheter: at the cranial end of the wound and the tip directed 4–5 cm cephalad to T1–4. The lower catheter was inserted at the caudal end of the wound, and the tip was directed 4–5 cm to a position at L1–4.	Ropivacaine 0.3% 4–8-ml boluses through each catheter.	Ropivacaine 0.3% 4–10 ml/h in each catheter after the bolus doses of ropivacaine	None
O’Hara [31] *Paediatr Anaesth* 2004 RCT 31	At the midpoint of the incision and advanced 3–5 cm cephalad	Bupivacaine 0.1% or 0.065% + fentanyl 5 μg/ml, 3 ml, before conclusion of surgery	Bupivacaine 0.1% or 0.065% + fentanyl 5 μg/ml, 4 ml/h, after the bolus of epidural solution before conclusion of surgery	None
Saudan [41] *Paediatr Anaesth* 2008 Observational 98	Single: the catheter tip was in the center of the surgical site. Double: the cranial catheter was set between thoracic levels T4 and T6 and the lower catheter between T10 and L1	Bupivacaine 0.25% with epinephrine 1:200,000 as a test dose after surgery. Bupivacaine 0.125%, 1.6 ml/kg as a loading dose in the absence of adverse events.	Bupivacaine 0.0625%, fentanyl 1 μg/ml, and clonidine 0.6 μg/ml, 0.2 ml/kg/h, in the recovery area	Doses of 0.1 ml/kg/h. Lockout time: 15 min, a maximal hourly bolus rate, 2
Gauger [20] *J Pediatr Orthop* 2009 RCT 38	At the midpoint of the incision and advanced 3–5 cm cephalad	Fentanyl 1 μg/kg and hydromorphone 5 μg/kg diluted in 0.3 ml/kg of saline, intraoperatively	Bupivacaine 0.1% + hydromorphone 10 μg/ml, 8 ml/h, on arrival in the PACU and after a neurologic examination	Bolus dose of 2 ml allowed every 30 min
Klatt [25] *Spine* (*Phila Pa 1976*) 2013 RCT 66	Single: at the midpoint of the incision and advanced 3–5 cm cephalad Double: the upper catheter was placed at the junction of the proximal and middle thirds of the instrumented spine and advanced 5 cm cephalad. The lower catheter was placed at the junction of the middle and distal thirds of the instrumented spine and advanced 5 cm cephalad.	Bupivacaine 0.1% and fentanyl 2 μg/ml, 0.2 ml/kg (single) or 0.1 ml/kg in each catheter (double), with initiation of wound closure	Bupivacaine 0.1% + fentanyl 2 μg/ml, 0.2 ml/kg/h (single) or bupivacaine 0.1% + fentanyl 2 μg/ml, 0.1 ml/kg/h in each catheter (double), upon arrival to the PACU	Demand dose of 0.1 ml/kg, lockout time 1 h (allowed only via the caudal catheter in the double catheter group)
Erdogan [43] *Spine* (*Phila Pa 1976*) 2017 RCT 44	At the midpoint of the incision and advanced 5–6 cm cephalad to T4 to T5	Morphine 50 μg/kg in a PCIEA group and 20 μg/kg in a PCCEA group	No continuous infusion in the PCIEA group and morphine 10 μg/kg/h in the PCCEA group	50 μg/kg/h with a 1-h lockout in PCIEA group and 5 μg/kg bolus dose with a 30-min lockout interval and 4-h limit of 4 mg

*PACU* post-anesthesia care unit, *PCEA* patient-controlled epidural analgesia, *PCEEA* patient-controlled continuous epidural analgesia, *PCIEA* patient-controlled intermittent bolus epidural analgesia, *RCT* randomized controlled trial

In summary, epidural analgesia is effective as a postoperative analgesia strategy and may provide better analgesia than IV PCA in patients undergoing PSF for AIS. However, it should be used with caution because there might be some drawbacks, such as the problem of inadequate assessment of motor function and the need to retain the urinary catheter.

### Intrathecal opioids

Intrathecal administration of opioids is another analgesic strategy [45–51]. In most reports, morphine 5–20 μg/kg alone or in combination with sufentanil 1 μg/kg and diluted in 2–4 ml of saline was administered intrathecally by an anesthesiologist after the induction of anesthesia and before the surgical incision. The results of these studies show that intrathecal opioids (ITO) can reduce intraoperative opioid use as well as early postoperative opioid consumption and pain scores, but the analgesic effect does not last for more than 24 h [21, 45–48, 50, 51] (Table 3). Therefore, this method is used in combination with IV PCA. It has been shown that ITO reduce intraoperative blood loss by 65% [45–47], although the mechanism of the blood-sparing effect of ITO is unclear. Some studies found that mean arterial pressure was lower in patients who received ITO than in those who did not, but other studies found no difference in the mean arterial pressure between these two groups. There was no difference in the frequency of nausea and vomiting (25.2–90%) or pruritus (4.3–80%) between patients who received ITO and those who received IV PCA alone. The incidence of clinically significant respiratory depression with ITO is reported to be similar to that with IV PCA, but a higher ITO dose (morphine ≥ 20 μg/kg) may increase the risk of clinically significant respiratory depression [48]. There are no reports of increased rates of spinal anesthesia-related complications, such as nerve injury, hematoma, or infection with ITO, but again, the possibility of these adverse events is not completely excluded because of the small sample sizes in most studies.

**Table 3:** Regimens for intrathecal opioids reported in the literature

First author Journal Year Type of study Total sample size	Timing of administration	Solution	Site	Needle
Goodarzi [45] *Paediatr Anaesth* 1998 RCT 80	After tracheal intubation	2 μg/kg morphine mixed with 50 μg of sufentanil and 2 ml of preservative-free saline	At the level of L3–4	A 24-gauge spinal needle
Gall [46] *Anesthesiology* 2001 RCT 30	After tracheal intubation	4-ml solution containing 2 μg/kg preservative-free morphine, 5 μg/kg preservative-free morphine, or normal saline	No description	A 25-gauge pencil-point spinal needle
Eschertzhuberl [47] *Br J Anaesth* 2008 RCT 42	After tracheal intubation	A mixture of 1 μg/kg sufentanil and 5 or 15 μg/kg morphine diluted with normal saline to a total volume of 3 ml	At the level of L3–4 or L4–5	A 25-gauge pencil-point spinal needle
Tripi [48] *Spine* (*Phila Pa 1976*) 2008 Retrospective 407	After induction of general anesthesia and before surgical incision	No dose, moderate dose; morphine 9–19 μg/kg or high dose ≥ 20 μg/kg	No description	No description
Son-Hing [49] *J Pediatr Orthop* 2011 Retrospective 287	After induction of general anesthesia and before surgical incision	Morphine 9–19 μg/kg	No description	No description
Hong [50] *Paediatr Anaesth* 2017 Retrospective 40	Immediately after induction of anesthesia and prior to incision	Morphine 12 μg/kg	No description	No description
Cohen [51] *Anesth Analg* 2017 RCT 71	After placement of an endotracheal tube	Preservative-free morphine at a concentration of 7.5 μg/kg	Between levels L2 and L5	A 24-gauge spinal needle

*RCT* randomized controlled trial

In summary, ITO is effective for the reduction of opioid consumption and pain intensity in the early postoperative period after scoliosis correction surgery. Further, ITO is associated with less intraoperative blood loss. However, a higher ITO dose (morphine ≥ 20 μg/kg) should be avoided in view of the potential risk of respiratory depression.

### Ketamine

Ketamine is an anesthetic agent that works by binding to the *N*-methyl-d-aspartate receptor and has an analgesic effect at subanesthetic doses. Recent meta-analyses have shown that supplemental perioperative ketamine decreases the intensity of postoperative pain and cumulative morphine consumption without increasing adverse events in patients undergoing various types of surgery [52, 53]. The recent guidelines recommend consideration of IV ketamine as a component of multimodal analgesia [34]. However, the postoperative analgesic effect of adjuvant ketamine in patients undergoing scoliosis correction surgery for AIS is controversial. Table 4 shows the regimens for continuous ketamine infusion reported in the literature. In one randomized controlled trial, 34 patients were assigned to receive either intraoperative low-dose ketamine (a bolus dose of 0.5 mg/kg followed by a continuous infusion of 0.24 mg/kg until extubation, *n* = 16) or the same volume of saline (*n* = 18) [30]. Intraoperative low-dose ketamine failed to decrease the cumulative morphine consumption, the pain scores, or the frequency of PONV and pruritus at 72 h after PSF when compared with the saline-treated group. In another randomized controlled trial, patients who received ketamine as a bolus dose of 0.5 mg/kg prior to the surgical incision, an intraoperative infusion at 0.25 mg/kg/h, and a postoperative infusion at 0.1 mg/kg/h for 72 h (*n* = 29) had morphine consumption and sedation and pain scores that were similar to those in a saline-treated group (*n* = 21) for 96 h postoperatively [28]. One randomized controlled trial failed to show beneficial effects of ketamine (administered as a 0.5 mg/kg bolus at induction of anesthesia followed by a continuous infusion of 2 μg/kg/min until 72 h postoperatively) on either short-term outcomes, i.e., postoperative morphine use, pain scores, and side effects, or the long-term outcome, i.e., incidence of pain after discharge from hospital [27]. In contrast, it has been reported that perioperative low-dose ketamine (a bolus dose of 0.5 mg/kg, followed by 48 h of continuous infusion of 0.12 mg/kg/h, *n* = 17) decreased the cumulative morphine consumption by 20% when compared with a control group (*n* = 19) at 24 and 48 h after surgery. In that study, pain scores, sedation scale scores, and the incidence of PONV did not differ between the two groups, but antiemetic consumption was lower in the ketamine group [54]. Ketamine-related adverse effects, such as neuropsychiatric symptoms (confusion, hallucinations, and nightmares) and cardiovascular events, have not been reported.

**Table 4:** Regimens for continuous ketamine infusion reported in the literature

First author Journal Year Type of study Total sample size	Loading dose	Intraoperative infusion	Postoperative infusion
Engelhardt [30] *Anaesth Analg* 2008 RCT 34	0.5 mg/kg (no description regarding the time of bolus administration)	4 μg/kg/min started after bolus dose and discontinued after surgery at the time of tracheal extubation	None
Pestieau [28] *Paediatr Anaesth* 2014 RCT 50	0.5 mg/kg immediately after the patient was positioned prone	0.25 mg/kg/h	0.1 mg/kg/h infused for 72 h
Minoshima [54] *Acta Anaesthesiol Scand* 2015 RCT 36	0.5 mg/kg after tracheal intubation	2 μg/kg/min started after loading dose	2 μg/kg/min for 48 h
Perello [27] *Spine* (*Phila Pa 1976*) 2017 RCT 44	0.5 mg/kg at induction of anesthesia	2 μg/kg/min	2 μg/kg/min for 72 h

*RCT* randomized controlled trial

In summary, there are insufficient data to support the routine use of ketamine as an adjuvant to postoperative analgesia in patients undergoing PSF for AIS. Further research is needed to determine the optimal timing, dose, and mode of the administration of ketamine when used for postoperative analgesia in these patients.

### Gabapentinoids

Gabapentin and pregabalin were originally developed as anticonvulsants and have been used widely to treat chronic neuropathic pain. Recent meta-analyses show that these agents are useful as adjuvant analgesia for reducing postoperative pain, including after spine surgery [55–57]. However, only the analgesic effect of gabapentin has been studied in scoliosis correction surgery (Table 5), and the results are inconsistent. In a randomized controlled trial that included 35 patients with AIS, a single dose of oral gabapentin (600 mg, 1 h before surgery, *n* = 18) did not reduce postoperative cumulative morphine consumption or pain intensity in the 72 h following surgery when compared with placebo (*n* = 17) [24]. In contrast, in another randomized controlled trial, multiple doses of gabapentin (15 mg/kg, 30 min before surgery and 5 mg/kg three times daily for 5 days postoperatively, *n* = 29) reduced the total morphine consumption in the recovery room and on postoperative days 1 and 2 by 30, 16, and 23%, respectively, in comparison with placebo (*n* = 30) [29]. Pain scores in the recovery room and on the morning after surgery were also reduced, but the differences did not reach statistical significance. In both studies, no difference in opioid-related side effects was found between the two groups. In a recent retrospective study, perioperative gabapentin (10 mg/kg 1 h before the arrival in the operating room and 200 mg three times a day starting on postoperative day 1 and continued until discharge from hospital) reduced the total morphine dose required by 25%. However, gabapentin did not reduce the pain scores during the postoperative stay or any side effects [26].

**Table 5:** Regimens for oral gabapentin reported in the literature

First author Journal Year Type of study *n*	Preoperative dose	Postoperative doses
Mayell [24] *Paediatr Anaesth* 2014 RCT 35	600 mg 1 h before surgery	None
Rusy [29] *Anaesth Analg* 2010 RCT 59	15 mg/kg 25–30 min before being taken to the OR	At a dose of 5 mg/kg three times/day, starting on POD 1 for 5 days
Choudhry [26] *J Pediatr Orthop* 2017 Retrospective 127	10 mg/kg up to a maximum of 600 mg 1 h before going to the OR	200 mg twice a day for patients > 50 kg body weight and 100 mg three times a day for patients < 50 kg body weight starting on POD 1 until discharge from hospital

*OR* operating room, *POD* postoperative day, *RCT* randomized controlled trial

In summary, gabapentin may have the potential to reduce pain after PSF in patients with AIS, but the optimal dosage and duration of administration are unclear. Furthermore, there is no strong evidence of the effectiveness of gabapentin as an analgesic adjuvant in this patient population, so further research is needed to confirm its efficacy.

### Acetaminophen/nonsteroidal anti-inflammatory drugs

Addition of scheduled acetaminophen and/or nonsteroidal anti-inflammatory drugs (NSAIDs) to opioid-based analgesia improves analgesia and reduces opioid requirements, so preemptive use of these agents in adults is included in the recommendations of several guidelines [33, 34]. The utility of these adjunctive analgesics in children is also supported by a recent systematic review [58]. However, few studies have examined the analgesic effects of acetaminophen/NSAIDs in patients undergoing scoliosis correction surgery (Table 6). In a randomized controlled trial, acetaminophen 90 mg/kg/day IV (30 mg/kg at the end of surgery, twice at 8-h intervals thereafter) reduced the number of patients with a visual analog scale (VAS) pain score of ≥ 6 within 24 h after surgery (7/18 patients in the acetaminophen group vs 13/18 patients in the placebo group) [32]. However, there was no difference in terms of the maximum VAS score (5.5 in the acetaminophen group vs 6.5 in the placebo group) or the cumulative oxycodone dose (1.3 ± 0.3 mg/kg in the acetaminophen group vs 1.43 ± 0.4 mg/kg in the placebo group) during the first 24 postoperative hours in that study. Some studies have evaluated the analgesic efficacy of NSAIDs in scoliosis correction surgery. Munro et al. showed that ketorolac 0.5 mg/kg administered at 6-h intervals postoperatively for 36 h reduced VAS pain scores on postoperative days 1 and 2 as well as morphine consumption in the post-anesthesia care unit and on postoperative day 2 when compared with placebo [17]. In a retrospective study that included 7349 patients undergoing PSF for AIS, the use of ketorolac was independently associated with significantly lower odds of a prolonged duration of IV opioids (odds ratio 0.84, 95% confidence interval 0.73–0.98) and a prolonged length of stay (odds ratio 0.75, 95% confidence interval 0.64–0.89) [59]. Given that NSAIDs exert their analgesic effects by preventing prostaglandin synthesis via inhibition of cyclooxygenase, there has been a concern regarding the effects of NSAIDs on bone formation and healing, which are critical processes for successful spinal fusion. Animal studies have shown that NSAIDs decrease the fusion rates after spinal fusion [60, 61]. Human studies have also shown that short-term exposure (for < 14 days) to normal doses of NSAIDs (ketorolac, diclofenac, celecoxib, and rofecoxib) was safe after spinal fusion, whereas high-dose ketorolac (≥ 120 mg/day) was associated with higher nonunion rates [62, 63]. Another potential concern regarding the use of perioperative NSAIDs is an increased risk of postoperative bleeding. Post-marketing surveillance data and a recent meta-analysis indicate that ketorolac does not increase postoperative blood loss [64, 65].

**Table 6:** Regimens for acetaminophen/nonsteroidal anti-inflammatory drugs reported in the literature

First author Journal Year Type of study Total sample size	Initial dose	Subsequent doses
Hiller [32] *Spine* (*Phila Pa 1976*) 2012 RCT 36	Acetaminophen 30 mg/kg IV for 15 min at the time of surgical closure	Subsequent doses of acetaminophen were given 8 and 16 h after the first dose
Munro [17] *Can J Anaesth* 2002 RCT 35	Ketorolac 0.5 mg/kg IV on completion of surgery	Repeat dosing of ketorolac every 6 h for a total of six doses
Rosenberg [59] *Spine* (*Phila Pa 1976*) 2016 Retrospective 7349	No description regarding dose and timing of ketorolac administration	No description regarding dose and timing of ketorolac administration

*IV* intravenous, *RCT* randomized controlled trial

In summary, acetaminophen and NSAIDs should be added as analgesic adjuvants unless contraindicated. High-dose ketorolac should be avoided because it may interfere with bone healing.

### Continuous wound infiltration

Continuous wound infiltration (CWI), whereby local anesthetics are administered continuously by an elastomeric pump via a catheter inserted at the surgical wound site, is being used increasingly as part of multimodal analgesia. In a recent meta-analysis comparing the analgesic effect of CWI with that of epidural analgesia in patients undergoing abdominal surgery, postoperative pain scores were similar between the two groups, and the incidence of urinary retention was significantly lower in the CWI group [66]. There are several studies suggesting the usefulness of CWI in scoliosis surgery (Table 7). In a retrospective study that compared patients who did (*n* = 129) and did not (*n* = 115) receive CWI, significantly fewer patients receiving CWI of bupivacaine 0.5% (4 ml/h for 100 h via a catheter placed adjacent to the spinal instrumentation, in the paraspinal muscles, in the subfascial area, or subcutaneously) required a continuous basal infusion of morphine (32.6% vs 85.2%), resulting in an overall reduction of opioid use on postoperative day 1 (18.9 mg vs 26.4 mg) [67]. In that study, the requirement for antiemetics was lower in patients who received CWI (91/129, 70.5%) than in those who did not (98/115, 85.2%). The depth of catheter placement did not impact the postoperative opioid use. In another retrospective study that again compared patients who did (*n* = 62) or did not (*n* = 25) receive CWI, CWI of bupivacaine 0.25% (4 ml/h for 100 h subcutaneously) decreased the opioid consumption by 38% within 24 h after scoliosis correction surgery [68]. There was no difference in the incidence of adverse effects, such as PONV, pruritus, and sedation, between the two groups. However, although no catheter-related complications were reported, there is a potential risk of infection through the catheter. In a retrospective study that evaluated the effectiveness of adjunctive pain control methods, a multivariate regression analysis showed that CWI reduced normalized opioid requirements by 0.98 mg/kg and reduced VAS pain scores by 0.67 points [23].

**Table 7:** Regimens for continuous wound infiltration of local anesthetics reported in the literature

First author Journal Year Type of study Total sample size	Position of the catheter	Loading dose	Continuous infusion
Ross [67] *Spine* (*Phila Pa 1976*) 2011 Retrospective 244	Just adjacent to the spinal instrumentation, in the paraspinal muscle, in the subfascial area, or subcutaneously at the discretion of the surgeon	None	Bupivacaine 0.5% at 4 ml/h for 100 h
Reynolds [68] *Global Spine J* 2013 Retrospective 87	Two catheters inserted into the subcutaneous tissue on either side of the incision site, just before wound closure	None	Bupivacaine 0.25% in sterile saline at a rateof 4 ml/h (2 ml/h for each catheter) for 100 h
Wade [23] *Spine Deform* 2015 Retrospective 196	Subcutaneously in the wound before closure	None	Bupivacaine 0.25% at 4 ml/h for 72 h

*RCT* randomized controlled trial

In summary, CWI seems to be a useful analgesic adjuvant in patients undergoing PSF for AIS. The currently available data are only from retrospective trials, so further prospective studies are needed to evaluate the safety and efficacy of CWI in this patient population.

### Diazepam

Diazepam is often used to prevent and treat muscle spasm after surgery for scoliosis. In general, diazepam is administered at a dose of 0.05–0.1 mg/kg every 6–12 h orally or IV as needed [20, 25, 28, 29, 32]. Although diazepam can suppress spasm, there is no evidence of its effect as an analgesic adjuvant [69].

### Other analgesics

Apart from the analgesics mentioned above, there are some agents that may improve analgesia, but they have not been studied in patients undergoing corrective surgery for AIS.

### Glucocorticoids

The efficacy of glucocorticoids for reducing pain and inflammation after surgery has been explored. A meta-analysis that included 5796 surgical patients demonstrated that a single IV dose of dexamethasone (1.25–20 mg) was associated with reductions in postoperative pain scores, opioid consumption, need for rescue analgesia, and length of stay in the post-anesthesia care unit, as well as a prolonged time to the first analgesic dose in comparison with the control group, without increasing the risks of infection or delayed wound healing [70]. Although dexamethasone has the potential to induce immunosuppression and increase the risk of infection, recent meta-analyses and a large clinical trial have shown that a single dose of dexamethasone does not increase the risk of surgical site infection in patients with or without diabetes mellitus undergoing noncardiac surgery [71, 72]. These data suggest that a single dose of dexamethasone could be an option for adjunctive analgesia in patients undergoing correction surgery for AIS. However, further studies are needed to establish the safety and efficacy of dexamethasone in this patient population, given that spinal instrumentation can increase the risk of soft tissue infections postoperatively [73].

### Intravenous lidocaine

IV lidocaine infusion has been shown to have analgesic and anti-inflammatory properties. A meta-analysis that included 2802 surgical patients showed that an IV infusion of lidocaine started at the time of surgery reduced the pain immediately after surgery (for up to 4 h) and until 24 h in patients undergoing laparoscopic or open abdominal surgery without increasing the risk of lidocaine-associated adverse effects, including death, arrhythmias, other cardiac conduction disorders, or signs of lidocaine toxicity [74]. Moreover, several studies have demonstrated that perioperative IV lidocaine decreases the incidence of chronic postoperative pain [75, 76], although the dose, timing, and duration of the administration of lidocaine varied between the studies. Thus far, no studies have examined the effect of IV lidocaine infusion on acute and chronic pain in patients with AIS undergoing PSF. Further research is needed to identify the optimal use of IV lidocaine infusion in this patient population.

### Prophylaxis of opioid-related side effects

As mentioned earlier, the high incidence of opioid-related adverse effects, particularly PONV and pruritus, is problematic in the management of postoperative pain in patients undergoing PSF for AIS. There are several measures that can minimize these complications. Prophylactic administration of antiemetics is the mainstay for the prevention of PONV. For patients at high risk for PONV, several antiemetics with different mechanisms of action may be needed, given that none of these agents alone has adequate prophylactic potency [77]. Dexamethasone and ondansetron are the most frequently used antiemetics in this patient population [12], although their effectiveness as antiemetics in pediatric surgery is only confirmed for minor procedures, such as tonsillectomy and strabismus surgery. There is no evidence of their antiemetic efficacy in major surgery, such as PSF for patients with AIS. Continuous administration of naloxone, an opioid antagonist, may be useful, given that this agent has been shown to reduce the incidence of pruritus and nausea without increasing the amount of analgesia needed [78, 79]. It has been reported that ondansetron may reduce opioid-induced pruritus [80]. Although there is no evidence of this as yet in pediatric cases, its use can be considered if needed.

### Persistent postsurgical pain in patients following scoliosis surgery

Patients undergoing PSF often develop persistent postoperative pain (PPP) in addition to acute pain in the immediate postoperative period. A retrospective survey reported that 55 (52%) of 105 patients had ongoing pain at the time of discharge from hospital [81]. In a recent prospective study of 144 adolescents who underwent scoliosis surgery, the incidence of pain was 37.8% at 2–3 months and 41.8% at 1 year after surgery [82]. The mechanism via which PPP develops is not fully elucidated but seems to involve neuropathic and even neuroinflammatory elements [83]. Severe acute postoperative pain has been proposed as one of the risk factors that might induce PPP [84]. Therefore, preemptive or preventive strategies that block the central sensitization mechanisms might be important for the prevention of PPP. The meta-analyses suggest that several techniques, including regional anesthesia, intravenous infusion of local anesthetics, and perioperative pregabalin and ketamine, may reduce PPP [57, 85, 86], but no promising results have been obtained as yet. The development of PPP appears to be multifactorial, so a multimodal approach might afford the best chance of favorable results.

## Conclusion

In summary, a potent analgesic technique such as IV PCA or epidural anesthesia is needed for pain management after PSF for AIS. Given the complex causes of pain and the high incidence of opioid-related complications, a multimodal approach to improve analgesia and reduce opioid consumption is essential. The efficacy of intrathecal opioids and NSAIDs as components of multimodal analgesia in PSF is well established. However, there is not enough evidence for the use of other analgesics or analgesic modalities in this patient population. PPP is also problematic in this patient population. Although several preventive strategies have been proposed, no promising results have been obtained. Further studies are needed to establish the safety and efficacy of these techniques and to identify the optimal multimodal regimen.

## Abbreviations

AIS: Adolescent idiopathic scoliosis
CWI: Continuous wound infiltration
ITO: Intrathecal opioids
IV PCA: Intravenous patient-controlled analgesia
NSAIDs: Nonsteroidal anti-inflammatory drugs
PONV: Postoperative nausea and vomiting
PPP: Persistent postsurgical pain
PSF: Posterior spinal fusion
VAS: Visual analog scale
